# Buprenorphine and postpartum contraception utilization among people with opioid use disorder: a multi-state analysis

**DOI:** 10.1186/s13722-024-00530-1

**Published:** 2025-01-06

**Authors:** Kevin Y. Xu, Jennifer K. Bello, Joanna Buss, Hendrée E. Jones, Laura J. Bierut, Dustin Stwalley, Hannah S. Szlyk, Caitlin E. Martin, Jeannie C. Kelly, Ebony B. Carter, Elizabeth E. Krans, Richard A. Grucza

**Affiliations:** 1 Health and Behavior Research Center, Division of Addiction Science, Prevention and Treatment, Department of Psychiatry, Renard Hospital 3007A, Washington University School of Medicine, 4940 Children’s Place, Saint Louis, MO 63110 USA; 2 Departments of Family and Community Medicine and Health and Clinical Outcomes Research, Saint Louis University School of Medicine, Saint Louis, MO USA; 3 Institute for Informatics, Department of Medicine, Washington University School of Medicine, Saint Louis, MO USA; 4 Department of Obstetrics and Gynecology, University of North Carolina School of Medicine, Chapel Hill, NC USA; 5 Department of Obstetrics and Gynecology, Virginia Commonwealth University School of Medicine, Richmond, VA USA; 6 Division of Maternal-Fetal Medicine & Ultrasound, Department of Obstetrics and Gynecology, Washington University School of Medicine, Saint Louis, MO USA; 7 Department of Obstetrics, Gynecology & Reproductive Sciences, Magee-Womens Research Institute, University of Pittsburgh School of Medicine, Pittsburgh, PA USA

**Keywords:** Contraception, LARC, Opioid use disorder, Buprenorphine, Administrative data

## Abstract

**Background:**

The postpartum period provides an opportunity for birthing people with opioid use disorder (OUD) to consider their future reproductive health goals. However, the relationship between the use of medication for opioid use disorder (MOUD) and contraception utilization is not well understood. We used multistate administrative claims data to compare contraception utilization rates among postpartum people with OUD initiating buprenorphine (BUP) versus no medication (psychosocial services receipt without MOUD (PSY)) in the United States (US).

**Methods:**

In this retrospective cohort study, we analyzed data from the Merative™ MarketScan^®^ Multi-State Medicaid Databases 2016–2021 among postpartum women with OUD who did and did not initiate BUP during pregnancy. Our primary outcome was the receipt of prescribed highly-effective or effective contraception by 90 days postpartum. Highly-effective contraception was defined as female sterilization and long-acting reversible contraception [LARC]). Effective contraception was defined as oral contraceptive pills [OCPs], the contraceptive patch, ring, or injection. We used multivariable Poisson regression models, adjusting for sociodemographic and clinical characteristics, to measure the association of BUP (vs. PSY) on postpartum contraception utilization.

**Results:**

Our sample consisted of 11,118 postpartum people with OUD. Among those, 3,443 initiated BUP and 7,675 received PSY. By 90 days postpartum, 22.4% (*n* = 2,487) of the cohort were prescribed contraception (21.5% PSY vs. 24.3% BUP). Among these participants, most received LARC (41.0%), followed by female sterilization (27.3%), the contraceptive injection (17.3%), pills (8.6%), ring (4.7%), and patch (1.0%), Compared to people engaged in PSY, BUP receipt was associated with a greater use of prescribed contraceptive use by 90 days postpartum (adjusted relative risk [aRR] = 1.17[1.07–1.28]), including a modestly greater use of the patch, ring, and pills, (aRR = 1.13[1.08–1.18]), but a modestly lesser use of injection contraception (aRR = 0.95[0.91–0.99]). There was no relationship observed between BUP and LARC use (aRR = 1.00[0.95–1.04]) and female sterilization (aRR = 1.01[0.98–1.06]).

**Conclusions:**

Only 22% of pregnant people with OUD in our cohort used effective or highly-effective postpartum contraception. BUP receipt during pregnancy, relative to PSY, was associated with modestly greater use of prescribed effective contraceptive methods but was not associated with greater use of provider-administered contraceptive methods, such as the contraceptive injection, LARC and female sterilization.

The online version contains supplementary material available at 10.1186/s13722-024-00530-1.

## Background

Few birthing people with opioid use disorder (OUD) receive postpartum contraception in the United States (U.S.) [1–3]. An analysis of postpartum Medicaid enrollees with OUD in Pennsylvania (2008–2013) found that only 25% received effective or highly-effective contraception, suggesting an unmet need for contraception access among birthing people with OUD [2]. Among Medicaid enrollees without OUD nationally, only one-third used an effective form of postpartum contraception in 2016 [4]. In recent years, there have been efforts by clinicians and policy makers to encourage prescribers of medication to treat OUD (MOUD: buprenorphine, naltrexone, and methadone) to provide easier access to contraception among reproductive-age people with OUD. In fact, the American College of Obstetricians and Gynecologists now recommends reproductive-age women with OUD to be routinely offered contraception [5] via a process of shared decision-making [6]. Integrating sexual and reproductive health services including provision of contraception with substance use treatment has commonly been hypothesized as a way to expand access to postpartum contraception [7–11]. However, there is a paucity of research that has evaluated the relationship between substance use treatment engagement, such as the initiation of MOUD, and the utilization of postpartum contraception.

Large, multi-state, administrative datasets can be particularly helpful for studying reproductive health outcomes during pregnancy due to their ability to provide a real-world, population level assessment of contraceptive utilization patterns. As a result, we used a large, multi-state Medicaid dataset to compare postpartum contraceptive utilization patterns among people initiating buprenorphine (BUP) versus those receiving psychosocial services *without* any MOUD use during pregnancy (PSY), which constitutes at least half of pregnant people with OUD [12, 13]. We specifically hypothesized that BUP receipt during pregnancy, as a proxy for greater engagement in substance use disorder (SUD) care, may be associated with greater contraception uptake postpartum in people with OUD compared to the receipt of PSY. Because contraception counseling is a shared decision-making process that balances efficacy and mechanism of action, among other factors such as cost and ease of access [6], we conducted subgroup analyses by contraception effectiveness (highly effective versus effective) and modality of receipt (prescribed versus provider-administered). There are numerous factors that influence contraceptive method choice including efficacy, side effects, cost, ease of access, and route and frequency of administration with the most effective methods requiring insertion by a provider while moderately effective methods being user-dependent and must be taken at certain intervals [6]. We thus aimed to explore if method effectiveness varied by treatment with MOUD versus psychosocial treatment alone due to the varying characteristics of methods by efficacy.

## Methods

### Data sources and cohort development

This study was a secondary analysis of a cohort of reproductive age females with OUD in the Merative™ MarketScan^®^ Multi-State Medicaid Databases (January 1, 2016–December 31, 2021) initiating BUP or PSY during pregnancy. The MarketScan databases include inpatient and ambulatory claims, as well as pharmacy claims from over 200 million unique people in the US. Each individual has a unique identifier that links people across years and file types (i.e., pharmacy claims, laboratory and diagnostic testing). Prior studies have raised concern for poor positive predictive values of standalone ICD codes for OUD; [14] our analyses, similar to prior analyses of MarketScan [12, 13, 15, 16], were limited to treatment-receiving individuals in an effort to increase the likelihood that our sample was correctly capturing individuals with active OUD (rather than a history of OUD). Further, patients had to have at least 7 months of insurance enrollment preceding either BUP or PSY initiation, thus identifying a group of patients engaged in healthcare services for the majority of a 9-month pregnancy. We calculated that more than 95% of the sample had at least 2 months of continuous insurance enrollment specifically during pregnancy, consistent with prior research [17]. 

**Fig. 1:**
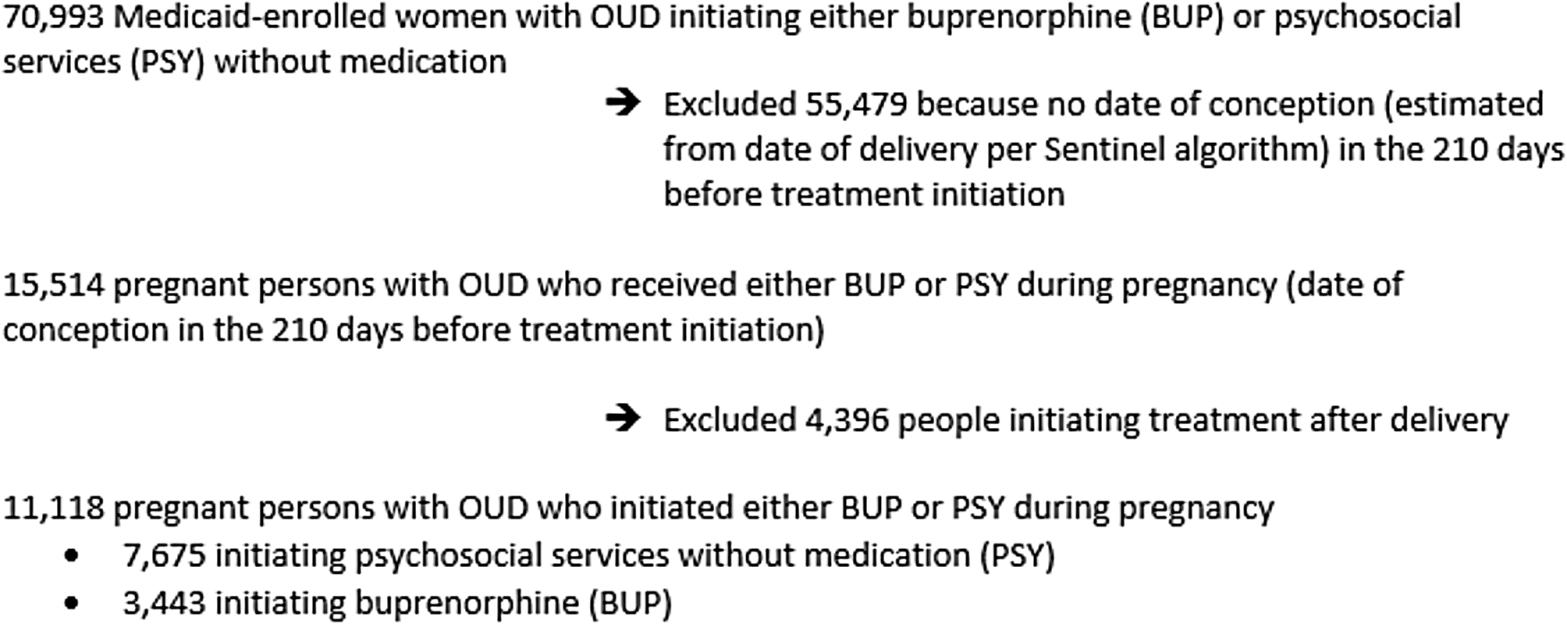
Derivation of the analytic sample

Among 70,993 Medicaid-enrolled women with OUD initiating either BUP or PSY, we identified 15,514 Medicaid-insured pregnant people with OUD who had a live birth and who initiated either BUP or PSY during pregnancy (Fig. 1). OUD was defined as persons who had International Statistical Classification of Diseases (ICD) -10 diagnosis of “opioid use, dependence, or abuse” (F11.XXX) occurring at least *twice* within the 7 months pre-index and 7 months post-index, in order to improve the specificity of OUD diagnostic codes. We used diagnosis and procedure codes to identify the date of delivery. An additional search for diagnosis and procedure codes within 7 days of delivery was implemented to estimate gestational age at the time of delivery via the Sentinel algorithm [18, 19]. If an estimate of gestational age could not be obtained using the Sentinel algorithm, pregnancy duration was set to 273 days as a default [18, 19]. People who initiated BUP or PSY after delivery (*n* = 4,396) were excluded, thus limiting our sample of participants to those who newly initiated BUP or PSY during pregnancy.

### Exposure variable: buprenorphine initiation vs. the receipt of psychosocial services without medication

Buprenorphine use (defined as > 1 prescription accompanied by a pharmacy fill) was identified using National Drug Codes (NDC) in outpatient pharmacy records linked to the MarketScan databases (Supplementary Information). Receipt of buprenorphine was coded as a binary variable, compared to the receipt of psychosocial services without any MOUD [PSY = reference group for individuals not receiving the standard of care “intervention” [20] for OUD]. The receipt of PSY were defined by ICD codes counseling, case management, and behavioral health services in any treatment setting (i.e., SUD treatment facilities, outpatient clinics). To increase the specificity of psychosocial services codes for OUD, we required at least one OUD diagnosis to be present on the psychosocial services claim. People receiving multiple forms of treatment simultaneously, defined as having the same dates associated with their medication claims (i.e., buprenorphine fills in pharmacy claims; procedure codes for naltrexone injections and methadone dispensing), were excluded (< 0.1% of the cohort).

### Outcome variable: postpartum contraception among treatment initiators

The primary outcome of interest was the binary variable of contraception receipt in the 90 days postpartum (yes versus no). All women were followed from 210 days prior to treatment initiation to 90 days after their date of delivery, which was selected as the post-delivery follow-up period because pregnancy-related Medicaid eligibility in the US typically provides coverage through a minimum of 12 weeks after delivery [21]. Contraceptive methods were further categorized into 4 groups for analysis: (1) no contraception (reference group) defined as the absence of highly-effective or effective contraceptive claims in our dataset, (2) LARC methods such as the Levonorgestrel-releasing IUD, the Copper T380A IUD and the subdermal contraceptive implant), (3) female sterilization and (4) user-dependent contraceptive methods which included the contraceptive injection (e.g., medroxyprogesterone acetate), the patch, ring, and pills [22]. Details on ascertainment of contraception are in the eTable 1. Contraception types were further grouped by effectiveness, differentiating between highly-effective (LARC; sterilization) and effective (patch, ring, OCPs, injection) methods. We also differentiated between provider-administered contraception (LARC; injection; sterilization) and contraception that can be filled at pharmacies (patch, ring, and OCPs). Covariates including demographics (age, race) and co-occurring disorders (psychiatric conditions, substance use disorders, and other medical conditions common in people with OUD such as insomnia, migraine, and chronic pain) in the 210 days preceding BUP or PSY initiation anticipated to approximately overlap with the pregnancy period were also extracted. ICD-10 and procedures codes used to define each category are described in the Supplementary Information. All data were de-identified, adherent to U.S. confidentiality requirements, and were determined to not involve human subjects research by the Washington University Human Research Protection Office.

### Statistical methods

We first conducted univariate analyses, depicting the association of demographic and clinical characteristics with contraception receipt using chi-square tests. To model the adjusted associations between OUD treatment type (BUP vs. PSY) and 90-day postpartum contraception uptake (any contraception vs. no contraception), we used modified Poisson regression, which are robust to outliers and non-linear confounders when estimating risk ratios (RR) for binary outcomes such as receipt of prescriptions [23]. Poisson models adjusted for demographic (age, race/ethnicity) and clinical comorbidities (co-occurring psychiatric disorders, substance use disorders, and medical comorbidities), as there is concern that co-occurring disorders may predict lower contraception uptake [24, 25]. After computing variance inflation factors to evaluate multicollinearity, we found no significant collinearity among all covariates (using a threshold of less than 2.0). We conducted several secondary analyses; first, we differentiated between contraception subtypes on a basis of effectiveness (highly effective vs. effective). Second, we differentiated between contraception subtypes based on their formulation (provider-administered versus prescribed).We conducted analyses using SAS 9.4. Our *p*-values were 2-sided. Statistical significance was set at *P* = .05. We analyzed data from March 14, 2023 (began deriving the original cohort) through March 22, 2024.

## Results

Our sample consisted of 11,118 postpartum, Medicaid-enrolled people identified on insurance claims as female, initiating BUP (31.0%) or PSY (69.0%). Overall, the mean age was 28.5 years (SD = 4.8), 60.4% were under the age of 30 years (59.9% PSY [mean age of 28.6 years] vs. 61.6% BUP [mean age of 28.4 years], *p* = .11) and 81.4% were White (83.9% PSY vs. 75.7% BUP) and 6.9% Black (7.3% vs. 6.0%, *p* < .001). Overall, the PSY group had a higher burden of co-occurring disorders compared to peers receiving BUP, including alcohol use disorder (PSY vs. BUP, 11.7% vs. 6.4%, *p* < .001), sedative use disorder (8.3% vs. 5.8%, *p* < .001), stimulant use disorder (29.2% vs. 17.5%, *p* < .001), anxiety disorder (41.6% vs. 34.2%, *p* < .001), mood disorder (40.1% vs. 31.3%, *p* < .001), and insomnia (5.3% vs. 3.8%, *p* < .001).

By 90 days postpartum, only 22.4% of our cohort had a claim for any contraception (21.5% PSY vs. 24.3% BUP, *p* < .001). Among those who received any contraception, the most common type was LARC (41.0%), followed by female sterilization (27.3%), the contraceptive injection (17.3%), pills (8.6%), ring (4.7%), and patch (1.0%), Among all individuals in the cohort, 9.2% received LARC, 6.1% sterilization, 3.9% injection, 1.9% pills, 1.1% ring, and 0.2% patch.

As illustrated in Table 1, among participants who received contraception by 90 days postpartum, those who received BUP were modestly less likely to receive LARC (9.6% PSY vs. 8.4% BUP) and the contraceptive injection (4.2% PSY vs. 3.1% BUP), but modestly more likely to receive the patch (0.1% PSY vs. 0.5% BUP), the vaginal ring (0.5% PSY vs. 2.2% BUP), and pills (0.9% PSY vs. 4.1% BUP).

**Table 1:** Postpartum contraception receipt after delivery stratified by buprenorphine (BUP) versus psychosocial services without medication (PSY)

	Overall	PSY, *N*(%)	BUP, *N*(%)		*P*-value
		7,675 (69.0)	3,443 (31.0)		
Age, mean (SD)	28.5 (4.8)	28.6 (4.7)	28.4 (4.8)		0.05
Age, Under 30 vs. *≥* 30	6,718 (60.4)	4,599 (59.9)	2,119 (61.6)		0.11
Race/Ethnicity					
Missing	832 (7.5)	474 (6.2)	358 (10.4)		< 0.001
Non-Hispanic White	9,049 (81.4)	6,442 (83.9)	2,607 (75.7)		
Non-Hispanic Black	766 (6.9)	558 (7.3)	208 (6.0)		
Hispanic	187 (1.7)	81 (1.1)	106 (3.1)		
Other	284 (2.6)	120 (1.6)	164 (4.8)		
Contraception Characteristics					
Any Contraception at 90 days Postpartum	2,487 (22.4)	1,649 (21.5)	838 (24.3)		< 0.001
Contraception Subtypes at 90 days					< 0.001
Prescription (effective)					
Patch	25 (0.2)	8 (0.1)	17 (0.5)		
Ring	118 (1.1)	41 (0.5)	77 (2.2)		
Oral contraceptive pills	213 (1.9)	71 (0.9)	142 (4.1)		
Provider-Administered (effective)					
Injection	430 (3.9)	324 (4.2)	106 (3.1)		
Provider-Administered (highly effective)					
Long-acting reversible contraception	1,022 (9.2)	734 (9.6)	288 (8.4)		
Female sterilization	679 (6.1)	471 (6.1)	208 (6.0)		
Co-Occurring Conditions					
Alcohol Use Disorder	1,115 (10.0)	894 (11.7)		221 (6.4)	< 0.001
Stimulant Use Disorder	2,845 (25.6)	2,244 (29.2)		601 (17.5)	< 0.001
Sedative Use Disorder	839 (7.6)	639 (8.3)		200 (5.8)	< 0.001
Anxiety Disorder	4,369 (39.3)	3,193 (41.6)		1,176 (34.2)	< 0.001
Mood Disorder	4,155 (37.4)	3,076 (40.1)		1,079 (31.3)	< 0.001
Insomnia	534 (4.8)	404 (5.3)		130 (3.8)	< 0.001
Migraine	395 (3.6)	289 (3.8)		106 (3.1)	< 0.001
Chronic Pain	568 (5.1)	350 (4.6)		218 (6.3)	< 0.001

In multivariable analyses adjusted for age, race, and co-occurring disorders, participants who received BUP were more likely to receive postpartum contraception at 90 days (adjusted relative risk [aRR] = 1.17[1.07–1.28]; Model 1 in Table 2, with full model in eTable 2). In subset analyses evaluating the effects of BUP receipt on highly-effective and effective contraception separately, BUP was modestly associated with the use of effective contraceptive methods at 90 days postpartum compared to PSY (aRR = 1.09[1.04–1.13]). Yet, no association was observed between BUP and LARC (aRR = 1.00[0.95–1.04]) and female sterilization uptake (aRR = 1.01[0.98–1.06]) relative to PSY (Model 2 in Table 2, full model in eTable 3).

**Table 2:** Adjusted analyses for contraception receipt 90 days after delivery

			Adjusted risk ratio [95% confidence intervals]
Model 1		Any contraception vs. no contraception	1.17 [1.07–1.28]
Model 2 Differentiating between highly-effective and effective subtypes	Highly-effective	LARC vs. no contraception	1.00 [0.95–1.04]
	Highly-effective	Female sterilization vs. no contraception	1.01 [0.98–1.06]
	Effective	Injection, OCP, patch, or ring vs. no contraception	1.09 [1.04–1.13]
Model 3 Differentiating between prescribed and provider-administered subtypes	Provider-administered	LARC vs. no contraception	1.00 [0.95–1.04]
	Provider-administered	Female sterilization vs. no contraception	1.01 [0.98–1.06]
	Provider-administered	Injection vs. no contraception	0.95 [0.91–0.99]
	Prescribed	OCP, patch, or ring vs. no contraception	1.13 [1.08–1.18]

Models above are controlling for age (< 30 vs. = > 30), race, (non-Hispanic Black vs. Hispanic vs. other vs. non-Hispanic White), and co-occurring disorders (alcohol/ stimulant /sedative use disorder; anxiety disorder; mood disorder; insomnia; migraine chronic pain). Full models can be found in the Supplementary Information

To evaluate how BUP receipt may differentially influence uptake of contraception based on location of receipt (provider-administered versus outpatient pharmacy prescription), we conducted an analysis that analyzed injections (provider-administered) separately from OCPs, patches, and rings (prescribed user-dependent). Whereas BUP, compared to PSY, was associated with a slightly lower use of injection receipt at 90-days postpartum (aRR = 0.95[0.91–0.99]), BUP was associated with a modestly greater use of prescription contraception use (aRR = 1.13[1.08–1.18]) (Model 3 in Table 2, full model in eTable 4).

## Discussion

In a multi-state cohort of pregnant people receiving treatment for OUD we found that 22% used contraception within the 3 months after delivery. Of the people receiving postpartum contraception, most received LARC or female sterilization, followed by female sterilization and injection. BUP was associated with modestly greater use of prescribed methods including the contraceptive pill, ring, and patch, but was not associated with greater use of provider-administered contraception such as LARC, female sterilization, and contraceptive injection. One potential implication of these data is that patients filling buprenorphine scripts may prefer prescribed contraception that is also available at pharmacies, as opposed to scheduling separate appointments for contraception that must be administered by a provider. Prior multi-state analyses have shown that expanding pharmacist prescriptions of contraception is associated with significantly improved contraception uptake [26]. A recent systematic review also suggested that most patients are interested in expanded access to contraception via pharmacies, which may be a function of their flexible hours relative to clinics and locations in proximity to the communities that they serve [27]. 

Even though LARC and female sterilization were the most common contraceptive methods used in our cohort, only 9.2% of all participants with OUD in our cohort received a LARC which is comparable to LARC utilization rates in the general population (10.4%) [28]. Whereas the uptake of LARC overall has more than doubled in the US since 2008 [29], such trends have not been observed in reproductive-age people with SUDs [3, 30], with many expressing strong preferences for barrier methods such as condoms [31]. While offering reproductive health services in SUD treatment settings has been suggested as a potential means to boost contraception uptake, particularly for highly-effective methods like LARC, studies have found that contraceptive uptake remains low for postpartum women with OUD even when prenatal and OUD care are integrated [7, 9, 32–34]. Studies evaluating pregnancy planning, inter-pregnancy interval, and contraceptive uptake have also not demonstrated a benefit to the co-location of reproductive health and substance use treatment services [7]. 

Despite their effectiveness and popularity among policymakers, highly-effective contraception uptake is hindered by mistrust between the health care system and people with a history of SUD (i.e., concerns about clinician pressure to use unwanted contraceptive methods; fear of clinician discrimination and forced sterilization [35]). In a study of people from historically marginalized groups at risk of preterm birth, a number of whom had a history of SUDs and trauma, individuals commonly described unmet information needs, discrimination, uncoordinated services, and stressful interactions with all levels of staff in the setting of contraception counseling [36]. In contrast, substance use treatment programs offering gender-specific programming (i.e., pregnancy-specific dosing, trauma-informed care, parenting skills training, child care, housing assistance, breastfeeding support) may be more effective at empowering patients to consider their reproductive autonomy [34]. Likewise, results from a recent clinical trial have demonstrated that a trauma-informed intervention (‘SAFE: Sex And Female Empowerment’), which supports people in OUD treatment through the contraception decision-making process, was associated with increased patient autonomy, attendance at a contraception consultation appointment, and LARC receipt [37].

Despite the study’s strengths, there are several important limitations. First, this is a treatment-receiving cohort; thus, this study is limited to persons with at least 7 months of insurance enrollment prior to initiating either BUP or PSY. As such, our findings cannot be generalized to persons with OUD who do not receive any treatment or are with limited or no insurance during their pregnancy. Second, this study was underpowered to assess contraception receipt among individuals receiving methadone or naltrexone, whose rates of pregnancy [38] and contraception receipt may differ from those receiving buprenorphine. Third, we did not account for parity, last menstrual period, and the use of non-prescribed contraception methods in the analyses. Non-prescription and over the counter contraceptive methods such as condoms, spermicides/sponges, withdrawal, Plan B, and natural family planning could not be identified in claims data and therefore, the use of these methods were not accounted for in analyses [1, 2]. Fourth, we did not control for differences by socioeconomic status, state or region, as this data was not available in the Medicaid subset of the MarketScan databases. The MarketScan Medicaid data is also limited in its level of detail for self-reported race and ethnicity, with prior work raising concern for poor ascertainment of Hispanic individuals despite recent efforts by administrative databases to collect data on race and ethnicity separately [13, 39]. Finally, while we hypothesized that buprenorphine treatment may be a proxy for greater engagement in SUD care, the modest association identified between BUP prescribing and contraception receipt may reflect person-level variables (i.e. motivations, attitudes, and preferences [35]) related to taking medications at a regular interval rather than the broader impact of BUP treatment on contraception access among a population of patients who are all engaged in treatment. Another limitation is the inability to account for medication discontinuation, which is common among pregnant people initiating BUP [12, 13].

In summary, we found that only approximately one-fifth of pregnant people initiating OUD treatment used effective or highly-effective postpartum contraception. While BUP receipt was associated with modestly greater use of *prescribed* effective contraceptive methods than PSY, it was not associated with greater use of provider-*administered* contraceptive methods, such as the contraceptive injection, LARC and female sterilization. While these data raise concern for gaps in contraception access, our findings also suggest that providing access to both SUD treatment and contraceptive methods in the same location may be a promising method to boost contraception uptake. Future investigations of contraceptive practices, desires, barriers and facilitators should incorporate evaluations guided by needs and preferences of people with lived experience.

## Electronic supplementary material

Below is the link to the electronic supplementary material.

Supplementary Material 1

Supplementary Material 2

## Data Availability

No datasets were generated or analysed during the current study.

## Declarations

### Dissemination declaration

Dissemination to study participants and or patient organizations is not possible or applicable due to the de-identified nature of our data.

### Presentations

Oral Presentation, AMERSA Annual Meeting, November 4 2023, Washington DC, USA
