# Correction: Buprenorphine and postpartum contraception utilization among people with opioid use disorder: a multi-state analysis

**DOI:** 10.1186/s13722-025-00556-z

**Published:** 2025-03-11

**Authors:** Kevin Y. Xu, Jennifer K. Bello, Joanna Buss, Hendrée E. Jones, Laura J. Bierut, Dustin Stwalley, Hannah S. Szlyk, Caitlin E. Martin, Jeannie C. Kelly, Ebony B. Carter, Elizabeth E. Krans, Richard A. Grucza

**Affiliations:** 1 Health and Behavior Research Center, Division of Addiction Science, Prevention and Treatment, Department of Psychiatry, Renard Hospital 3007A, Washington University School of Medicine, 4940 Children’s Place, Saint Louis, MO 63110 USA; 2 Departments of Family and Community Medicine and Health and Clinical Outcomes Research, Saint Louis University School of Medicine, Saint Louis, MO USA; 3 Institute for Informatics, Department of Medicine, Washington University School of Medicine, Saint Louis, MO USA; 4 Department of Obstetrics and Gynecology, University of North Carolina School of Medicine, Chapel Hill, NC USA; 5 Department of Obstetrics and Gynecology, Virginia Commonwealth University School of Medicine, Richmond, VA USA; 6 Division of Maternal-Fetal Medicine & Ultrasound, Department of Obstetrics and Gynecology, Washington University School of Medicine, Saint Louis, MO USA; 7 Department of Obstetrics, Gynecology & Reproductive Sciences, Magee-Womens Research Institute, University of Pittsburgh School of Medicine, Pittsburgh, PA USA

**Correction: Addict Sci Clin Pract 20, 1 (2025)**

10.1186/s13722-024-00530-1

Following publication of the original article [1], the authors noticed an error in the language used to describe the cohort development protocol, as well as the associated Fig. 1.

In the **Data sources and cohort development** section, one sentence is changed from:

“People who initiated BUP or PSY before pregnancy (*n* = 4,396) were excluded, thus limiting our sample of participants to those who newly initiated BUP or PSY during pregnancy.”

To:

People who initiated BUP or PSY after delivery (*n* = 4,396) were excluded, thus limiting our sample of participants to those who newly initiated BUP or PSY during pregnancy.

Fig. 1 is changed from:

**Fig. 1:**
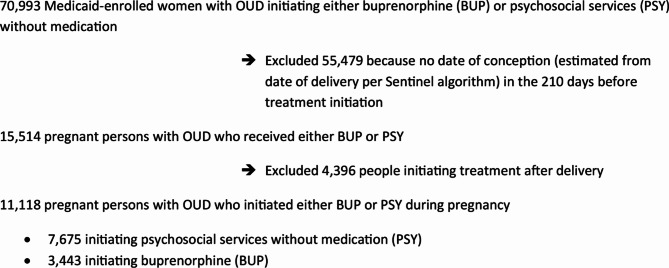
Derivation of the analytic sample

To:

**Fig. 1:**
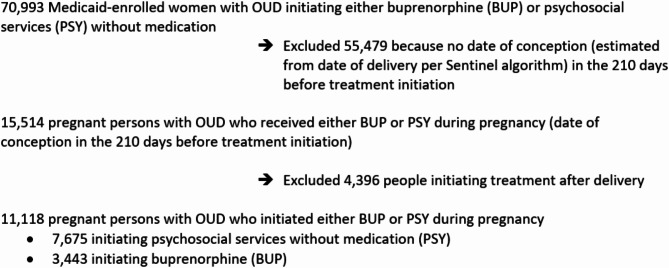
Derivation of the analytic sample

The authors apologise for this error and regret any inconvenience caused.

The original article [1] has been updated.

## References

[1] Xu KY, Bello JK, Buss J, et al. Buprenorphine and postpartum contraception utilization among people with opioid use disorder: a multi-state analysis. Addict Sci Clin Pract. 2025;20:1. 10.1186/s13722-024-00530-1. 39762993 10.1186/s13722-024-00530-1 PMC11702041

