# Supplementary material for: Buprenorphine and postpartum contraception utilization among people with opioid use disorder: a multi-state analysis
Source: Addict Sci Clin Pract. 2025 Jan 6;20:1. doi: 10.1186/s13722-024-00530-1 (PMC11702041; doi:10.1186/s13722-024-00530-1)
Supplement: Supplementary file 2 — Supplementary Material 2 [file 13722_2024_530_MOESM2_ESM.docx]

Online-Only Supplement

**eTable 1-** Supplementary Table depicting the coding of key variables in the analysis

| Variable | MarketScan |
| --- | --- |
| Pregnancy | **ICD or CPT codes**: 01960, 01961, 01962, 01963, 01967, 01968, 01969, 59050, 59051, 59400, 59409, 59410, 59412, 59414, 59430, 59510, 59514, 59515, 59525, 59610 59612, 59614, 59618, 59620, 59622, 99436, 99440, 10D0, 10E0, Z37, O80, O82  **ICD codes for preterm deliveries**: O60, P07, O42 |
| Contraception | **Patch via generic name variable (GENNME):** NORELGESTROMIN  **Oral contraception** (**GENNME**): DESOGESTREL, ETHYNODIOL, DROSPIRENONE, NORGESTREL, DIENOGEST, or NORGESTIMATE  **Nuvaring via generic name (GENNME):** ETONOGESTREL  **Annovera via product name search (PRODNME):** NESTORONE OR ELCOMETRINE or ANNOVERA  **Injection**/**Depo provera (NDC Number):** 00009470913  **LARC-IN CPT:** 11981 , 58300  **LARC-OUT CPT**: 11982, 11983 , 58301  **IUD CPT**: 58300, 58301  **Implant CPT:** 11981, 11982, 11983  **Female Sterilization** **CPT**: A4264, 58565, 58600, 58605, 58611, 58615, 58670, 58671  **Female Sterilization ICD codes**: 0U574ZZ, 0U578ZZ, 0UL74CZ, 0UL74DZ, 0UL74ZZ, 0UL78DZ, 0UL78ZZ |
| Diagnosis of “Opioid Use, Dependence, or Abuse” | ICD: F11.xxx |
| Initiation of Treatment for OUD | **NDC (national drug code) for buprenorphine**: 00054017613, 00054017713, 00054018813, 00054018913, 00093537856, 00093537956 ,00093572056, 00093572156, 00228315303, 00228315403, 00228315473, 00228315503, 00228315573, 00228315603, 00378092393, 00378092493, 00406192303, 00406192403, 00490005130, 12496120201, 12496120203, 12496120401, 12496120403, 12496120801, 12496120803, 12496121201, 12496121203, 12496127802, 12496128302, 12496130602, 12496131002, 16590066630, 16590066730, 23490927003, 35356000430, 38779088800, 38779088801, 38779088803, 38779088809, 42291017430, 42291017530, 43063018430, 49452129203, 49452825301, 49452825302, 49452825303, 49452825304, 49999039515, 49999063830, 49999063930, 50383028793, 50383029493, 50383092493, 50383093093, 51927101200, 52959030430, 52959074930, 54123011430, 54123091430, 54123092930, 54123095730, 54123098630, 54569573900, 54569573901, 54569573902, 54569639900, 54868570700, 54868570701, 54868575000, 55700014730, 59385001201, 59385001230, 59385001401, 59385001430, 59385001601, 59385001630, 60429058630, 60429058730, 62991158301, 62991158302, 62991158303, 62991158304, 63275992202, 63370090506, 63370090509, 63370090510, 63370090515, 63874108503, 63874117303, 65162041503, 65162041603, 68071151003, 68308020830  **Psychosocial Services without Medication to Treat OUD:**  HCPCS : H2021, H2022, H2023, H2024, H2025, H2026, H2027, H2028, H2029, H2030, H2031, H2032, H2033, H2034, H2035, H2036, H2037, T1002, T1006, T1007 T1012, T1016, T2048  CPT:90791, 90832, 90834, 90837, 90840, 90845, 90846, 90847, 90849, 90853, 90839, 90801, 90802, 90804, 90806, 90808, 90810, 90812, 90814, 90816, 90818, 90821, 90823, 90826 90828 90857,  ICD: HZ30ZZZ, HZ31ZZZ, HZ32ZZZ, HZ33ZZZ, HZ34ZZZ, HZ35ZZZ, HZ36ZZZ, HZ37ZZZ, HZ38ZZZ, HZ39ZZZ, HZ3BZZZ, HZ40ZZZ, HZ41ZZZ, HZ42ZZZ, HZ43ZZZ, HZ44ZZZ, HZ45ZZZ, HZ46ZZZ, HZ47ZZZ, HZ48ZZZ, HZ49ZZZ, HZ4BZZZ  **Naltrexone:**  Oral naltrexone was identified via the GENNME (generic name) query of “NALTREXONE” in the MarketScan prescription files. Extended-release naltrexone injections were identified using HCPCS code, J2315.  **Methadone:**  HCPCS: H2020  Methadone for chronic pain is EXCLUDED in the HCPCS procedure code, as it is ascertained via medication scripts (national drug codes), allowing us to restrict methadone services to those for the treatment of opioid use, dependence, or abuse  Note: Naltrexone and methadone claims were initially pulled in order to curate a sample of people with OUD initiating treatment. However, they were excluded from the final analytic sample (see Figure 1). |
| Age | Age_at_ep_Start |
| Alcohol Use Disorder | ICD: F10 |
| Stimulant Use Disorder  (Amphetamine or Cocaine Use Disorder | ICD: F14 F15 |
| Sedative Use Disorder | ICD: F13 |
| Anxiety Disorders | ICD: F40-F49 |
| Mood Disorders | ICD: F30-F39, encompassing major depressive disorder or bipolar disorders |
| Insomnia | ICD G47, F51 |
| Migraine | ICD: G43 |
| Chronic Pain | ICD: G89.2 |

**eTable 2:** Contraception Receipt 90 Days After Delivery (Any contraception vs no contraception)

|  | **Receipt of any contraception (vs no contraception)** | | |
| --- | --- | --- | --- |
|  | Adjusted relative (aRR) | 95% Confidence interval (CI) | |
| Buprenorphine vs Psychosocial Services without medication to treat OUD | 1.17 | 1.07 | 1.28 |
| Age, < 30 vs => 30 years | 1.01 | 0.93 | 1.10 |
| Non-Hispanic Black v Non-Hispanic White | 0.94 | 0.80 | 1.10 |
| Hispanic vs Non-Hispanic White | 1.02 | 0.76 | 1.37 |
| Other Race v Non-Hispanic White | 0.83 | 0.64 | 1.09 |
| Co-occurring alcohol, sedative, or stimulant use disorder | 1.02 | 0.93 | 1.12 |
| Anxiety vs No Anxiety Disorder | 1.00 | 0.91 | 1.10 |
| Mood vs No Mood Disorder | 1.06 | 0.88 | 1.27 |
| Insomnia vs No Insomnia | 1.00 | 0.82 | 1.21 |
| Migraine vs No Migraine | 1.05 | 0.84 | 1.31 |
| Chronic Pain vs No Chronic Pain | 1.02 | 0.92 | 1.12 |

**eTable 3**: Contraception Receipt 90 Days After Delivery, differentiating between highly effective and effective contraception

|  | **Highly-Effective** Contraception | | | | | | **Effective** User-Dependent Contraception | | |
| --- | --- | --- | --- | --- | --- | --- | --- | --- | --- |
|  | Receiving LARC vs no contraception | | | Receiving female sterilization vs no contraception | | | Receiving injection / OCP /patch/ring vs no contraception | | |
|  | aRR | 95% CI | | aRR | 95% CI | | aRR | 95% CI | |
| BUP v PSY | 1.00 | 0.95 | 1.04 | 1.01 | 0.98 | 1.06 | 1.09 | 1.04 | 1.13 |
| Age, < 30 vs => 30 | 0.99 | 0.95 | 1.03 | 1.19 | 1.14 | 1.23 | 0.96 | 0.93 | 1.00 |
| NH Black v NH White | 1.00 | 0.92 | 1.07 | 0.90 | 0.84 | 0.97 | 1.02 | 0.95 | 1.09 |
| Hispanic vs NH White | 1.04 | 0.90 | 1.20 | 0.83 | 0.71 | 0.96 | 1.01 | 0.87 | 1.17 |
| Other Race v NH White | 0.98 | 0.87 | 1.11 | 0.83 | 0.74 | 0.93 | 1.01 | 0.90 | 1.13 |
| Co-occurring AUD, StimUD, or SedUD | 1.02 | 0.98 | 1.06 | 0.94 | 0.91 | 0.98 | 1.00 | 0.96 | 1.04 |
| Anxiety vs No Anxiety D/o | 1.00 | 0.95 | 1.05 | 0.98 | 0.95 | 1.03 | 1.01 | 0.96 | 1.05 |
| Mood vs No Mood D/o | 0.99 | 0.90 | 1.09 | 1.11 | 1.03 | 1.20 | 1.00 | 0.92 | 1.10 |
| Insomnia vs No Insomnia | 1.01 | 0.92 | 1.11 | 0.95 | 0.87 | 1.04 | 0.99 | 0.90 | 1.09 |
| Migraine vs No Migraine | 1.00 | 0.89 | 1.11 | 0.98 | 0.89 | 1.08 | 1.04 | 0.94 | 1.16 |
| Chronic Pain vs No Chronic Pain | 1.01 | 0.96 | 1.06 | 1.04 | 1.00 | 1.08 | 0.98 | 0.93 | 1.02 |

AUD=alcohol use disorder, BUP=buprenorphine, CI=confidence interval, D/o=Disorder, LARC=long-acting reversible contraception, NH=Non-Hispanic, OCP=oral contraceptive pills, OR=odds ratio, PSY=psychosocial services without medication to treat opioid use disorder, SedUD=sedative use disorder; StimUD=stimulant use disorder

**eTable 4:** Contraception Receipt 90 Days After Delivery, differentiating between provider-administered contraception and prescribed contraception

|  | **Highly-Effective** Contraception – Provider-Administered | | | | | | **Effective** Contraception - Provider-Administered | | | **Effective** Contraception - Obtained via **Pharmacy** | | |
| --- | --- | --- | --- | --- | --- | --- | --- | --- | --- | --- | --- | --- |
|  | Receiving LARC vs no contraception | | | Receiving female sterilization vs no contraception | | | Receiving injection vs no contraception | | | Receiving OCP/ patch/ring vs no contraception | | |
|  | aRR | 95% CI | | aRR | 95% CI | | aRR | 95% CI | | aRR | 95% CI | |
| BUP v PSY | 1.00 | 0.95 | 1.04 | 1.01 | 0.98 | 1.06 | 0.95 | 0.91 | 0.99 | 1.13 | 1.08 | 1.18 |
| Age, < 30 vs => 30 | 0.99 | 0.95 | 1.03 | 1.19 | 1.14 | 1.23 | 0.98 | 0.94 | 1.02 | 0.97 | 0.93 | 1.01 |
| NH Black v NH White | 1.00 | 0.92 | 1.07 | 0.90 | 0.84 | 0.97 | 1.05 | 0.98 | 1.13 | 1.00 | 0.92 | 1.08 |
| Hispanic vs NH White | 1.04 | 0.90 | 1.20 | 0.83 | 0.71 | 0.96 | 1.11 | 0.97 | 1.28 | 0.96 | 0.82 | 1.13 |
| Other Race v NH White | 0.98 | 0.87 | 1.11 | 0.83 | 0.74 | 0.93 | 0.92 | 0.81 | 1.04 | 1.04 | 0.92 | 1.17 |
| Co-occurring AUD, StimUD, or SedUD | 1.02 | 0.98 | 1.06 | 0.94 | 0.91 | 0.98 | 1.02 | 0.98 | 1.06 | 0.99 | 0.94 | 1.04 |
| Anxiety vs No Anxiety D/o | 1.00 | 0.95 | 1.05 | 0.98 | 0.95 | 1.03 | 1.01 | 0.97 | 1.06 | 1.00 | 0.96 | 1.05 |
| Mood vs No Mood D/o | 0.99 | 0.90 | 1.09 | 1.11 | 1.03 | 1.20 | 1.02 | 0.94 | 1.12 | 0.99 | 0.90 | 1.10 |
| Insomnia vs No Insomnia | 1.01 | 0.92 | 1.11 | 0.95 | 0.87 | 1.04 | 0.97 | 0.89 | 1.07 | 1.00 | 0.91 | 1.11 |
| Migraine vs No Migraine | 1.00 | 0.89 | 1.11 | 0.98 | 0.89 | 1.08 | 1.14 | 1.04 | 1.26 | 0.98 | 0.87 | 1.10 |
| Chronic Pain vs No Chronic Pain | 1.01 | 0.96 | 1.06 | 1.04 | 1.00 | 1.08 | 0.96 | 0.91 | 1.00 | 0.99 | 0.95 | 1.04 |

AUD=alcohol use disorder, BUP=buprenorphine, CI=confidence interval, D/o=Disorder, LARC=long-acting reversible contraception, NH=Non-Hispanic, OCP=oral contraceptive pills, OR=odds ratio, PSY=psychosocial services without medication to treat opioid use disorder, SedUD=sedative use disorder; StimUD=stimulant use disorder
